# Cefepime shows good efficacy and no antibiotic resistance in pneumonia caused by *Serratia marcescens* and *Proteus mirabilis* – an observational study

**DOI:** 10.1186/s40360-016-0056-y

**Published:** 2016-03-23

**Authors:** Josef Yayan, Beniam Ghebremedhin, Kurt Rasche

**Affiliations:** Department of Internal Medicine, Division of Pulmonary, Allergy and Sleep Medicine, HELIOS Clinic Wuppertal, Witten/Herdecke University, Witten, Germany; Witten/Herdecke University, Institute of Medical Laboratory Diagnostics, Center for Clinical and Translational Research, HELIOS Clinic Wuppertal, Witten, Germany

**Keywords:** *Serratia marcescens*, *Proteus mirabilis*, Cefepime, Pneumonia, Antibiotic, Sensitivity, Resistance

## Abstract

**Background:**

Many antibiotics have no effect on Gram-positive and Gram-negative microbes, which necessitates the prescription of broad-spectrum antimicrobial agents that can lead to increased risk of antibiotic resistance. These pathogens constitute a further threat because they are also resistant to numerous beta-lactam antibiotics, as well as other antibiotic groups. This study retrospectively investigates antimicrobial resistance in hospitalized patients suffering from pneumonia triggered by Gram-negative *Serratia marcescens* or *Proteus mirabilis*.

**Methods:**

The demographic and clinical data analyzed in this study were obtained from the clinical databank of the HELIOS Clinic, Witten/Herdecke University, Wuppertal, Germany, for inpatients presenting with pneumonia triggered by *S. marcescens* or *P. mirabilis* from 2004 to 2014. An antibiogram was conducted for the antibiotics utilized as part of the management of patients with pneumonia triggered by these two pathogens.

**Results:**

Pneumonia was caused by Gram-negative bacteria in 115 patients during the study period from January 1, 2004, to August 12, 2014. Of these, 43 (37.4 %) hospitalized patients [26 males (60.5 %, 95 % CI 45.9 %–75.1 %) and 17 females (39.5 %, 95 % CI 24.9 %–54.1 %)] with mean age of 66.2 ± 13.4 years had pneumonia triggered by *S. marcescens*, while 20 (17.4 %) patients [14 males (70 %, 95 % CI 49.9 %–90.1 %) and 6 females (30 %, 95 % CI 9.9 %–50.1 %)] with a mean age of 64.6 ± 12.8 years had pneumonia caused by *P. mirabilis. S. marcescens* showed an increased antibiotic resistance to ampicillin (100 %), ampicillin-sulbactam (100 %), and cefuroxime (100 %). *P. mirabilis* had a high resistance to tetracycline (100 %) and ampicillin (55 %). *S. marcescens* (*P* < 0.0001) and *P. mirabilis* (*P* = 0.0003) demonstrated no resistance to cefepime in these patients with pneumonia.

**Conclusions:**

*S. marcescens* and *P. mirabilis* were resistant to several commonly used antimicrobial agents, but showed no resistance to cefepime.

## Background

*Serratia marcescens* and *Proteus mirabilis* are Gram-negative bacteria that can cause pneumonia—an acute infection of the lower airways caused by airborne infection or by infection transferred from another part of the body via the bloodstream [1]. Gram-negative bacteria are less often the cause of community- and nosocomial-acquired pneumonia when compared to Gram-positive bacteria [2–4].

*S. marcescens* is the most common species of the *Enterobacteriaceae* family responsible for nosocomial infections [5, 6]. *Proteus* species are other Gram-negative bacteria from the *Enterobacteriaceae* family [7], with *P. mirabilis* being a medically prominent species [8]. *Proteus* spp. cause most of the common urinary tract infections and are less frequently the causes of infections in other locations, including pneumonia [8].

Penicillin has reliably helped to treat potentially fatal bacterial infections for many decades [9]. However, antibiotics have lost their effectiveness due to the increasing antibiotic resistance expressed by microbes [10]. Many antibiotic resistant Gram-positive pathogens are recognized, but antibiotic resistance is also becoming increasingly common in Gram-negative bacteria [11]. Gram-positive and Gram-negative microorganisms often can only be fought with different antibiotics, making the timely use of effective antibiotics particularly important in patients with pneumonia.

Early identification of specific resistance characteristics can result in a more effective use of antibiotics and can limit the use of broad-spectrum antibiotics to serious infections, thereby helping to prevent the development of antibiotic resistance in *S. marcescens* and *P. mirabilis*. Early isolation of infected patients can also help to stop the spread of resistant bacteria.

For these reasons, this research was performed to identify the antibiotics to which *S. marcescens* and *P. mirabilis* have shown resistance over the past 10 years. The records of the HELIOS Clinic at Witten/Herdecke University in Wuppertal, Germany were searched to gather all relevant files on hospitalized patients suffering from pneumonia triggered by *S. marcescens* and *P. mirabilis* and classified according to the International Classification of Diseases (ICD) code J15.6 [12, 13]. The goal of this investigation was to determine the most effective selection of active antibiotics against *S. marcescens* and *P. mirabilis*, in order to reduce the suffering of patients, shorten the duration of hospital stays, and decrease the number of deaths.

## Methods

### Patients

This retrospective observational study analyzed antibiotic resistance in all hospitalized patients over the age of 18 years with identified pneumonia triggered by *S. marcescens* or *P. mirabilis*. A parallel evaluation was made of the antibiotic sensitivity and resistance of patients with pneumonia caused by *S. marcescens* and with pneumonia due to *P. mirabilis*. All appropriate data were acquired from files in the hospital databank of the HELIOS Clinic at Witten/Herdecke University in Wuppertal, Germany, for the duration of this investigation from January 1, 2004, to August 12, 2014.

### Definition of pneumonia

Pneumonia is an acute inflammation of the lower airways that can be triggered by *S. marcescens* or *P. mirabilis*. The characteristic clinical signs of pneumonia are productive cough, chest pain, fever, and shortness of breath. Pneumonia is identified by chest X-ray investigation and expectorant cultures [12, 13].

Community-acquired pneumonia triggered by *S. marcescens* or *P. mirabilis* is an acute respiratory tract infection picked up from ordinary communal interaction with the public; this differs from nosocomial-acquired pneumonia triggered by *S. marcescens* or *P. mirabilis*, which occurs during hospitalization [14].

After a first empirical antibacterial treatment, the diagnosis of pneumonia caused by *S. marcescens* or *P. mirabilis* was based on the specific criteria that all patients were hospitalized, all presented new areas of infiltration on X-ray investigation, and all had novel clinical symptoms, including a minimum of two of the following symptoms: difficulty breathing, fever over 38 °C, sputum production, and coughing.

### Investigated antibiotics

The effectiveness of the following antibiotics was examined against *S. marcescens* and *P. mirabilis* by susceptibility testing: ampicillin, piperacillin, ampicillin-sulbactam, cefotaxime, cefuroxime, piperacillin-tazobactam, ceftazidime, cefepime, meropenem, tobramycin, imipenem, levofloxacin, ciprofloxacin, gentamicin, co-trimoxazole, tetracycline, tigecycline, amikacin, and fosfomycin.

The incidence was noted of the application of the above antimicrobial agents in clinical use for the therapy of inpatients suffering from pneumonia triggered by *S. marcescens* or *P. mirabilis*. The rate of susceptibility testing of these antibiotics after discovery of *S. marcescens* or *P. mirabilis* was also recorded. *S. marcescens* and *P. mirabilis* causing pneumonia were assessed for susceptibility to antibiotics and then the antimicrobial agent showing the greatest resistance was checked against the other antibiotics.

For Gram-negative pathogens, minimal inhibitory concentration (MIC) breakpoints were utilized that corresponded to the antibiotic susceptibility testing guidelines established by the Clinical and Laboratory Standards Institute (CLSI) for 2004 – 2011 [15], and by the European Committee on Antimicrobial Susceptibility Testing (EUCAST) for 2012 – 2014 [16].

### Detection and antimicrobial susceptibility testing

Gram-negative bacteria were identified based on growth on chocolate agar that included bacitracin (BD™ MacConkey Agar, Becton Dickinson, Heidelberg) after incubation for 18–48 h at 37 °C with 5 % CO_2_. These bacteria were identified as oxidase-positive, porphyrin-negative bacteria needing nicotinamide adenine dinucleotide plus heme in addition to missing beta-hemolysis on horse blood agar plates, and by MALDI-TOF-MS (Matrix Assisted Laser Desorption Ionization Time-of-Flight-Mass Spectrometry, Bruker, Bremen, Germany). Software suitable for the interpretation of susceptibility testing results using the EUCAST breakpoints for 2012 – 2014 was utilized for the antimicrobial susceptibility testing [16]. Gram-negative isolates were additionally examined using the API NH biochemical reaction technique for the detection of *Neisseria* and *Haemophilus* species (bioMérieux, Marcy-l’Étoile, France) and by MALDI-TOF MS analysis (Daltonik, Bremen, Germany).

### EUCAST standardized disc diffusion method

The disc diffusion method established by Kirby-Bauer was carried out for antimicrobial susceptibility testing [17]. Mueller-Hinton culture medium was supplemented with 5 % horse blood and 20 mg/L beta-nicotinamide adenine dinucleotide (BD, Heidelberg, Germany). Plates were inoculated with samples of each isolate and set to a turbidity of 0.5 McFarland. Antibiotic discs were applied to the dried surface of the inoculated culture medium and later incubated at 35 ± 1 °C for 18 ± 2 h in a 5 % CO_2_ atmosphere. In cases of discrepancies or insufficient readings, the accurate determination of the MIC was executed by an E-test for particular pathogens, and the outcomes were interpreted according to the EUCAST criteria [16]. Intermediate isolates were grouped together with resistant isolates. Beta-lactamase production was evaluated using the nitrocefin test (Oxoid, Wesel, Germany). Gram-negative strains were described as beta-lactamase-negative strains that were resistant to ampicillin (zone diameter > 16 mm or MIC ≥ 4 μg/mL) [16]. Inhibition zone diameters were based on the 2014 EUCAST guidelines [16].

### Microbiology

Bronchoalveolar lavage using fiber-optic video bronchoscopy was performed for microbiological examination of secretions from the pulmonary airways. After administration of local anesthesia, approximately 20 ml of isotonic saline was administered and aspirated by means of the fiber-optic bronchoscope into three special sterilized 40-ml sample containers. In this way, tracheal secretions were also obtained by means of bronchoscopy. Throat smears were obtained by turning a sterile cotton swab (MEUS Srl, Piove di Sacco, Italy) along the throats of inpatients presumed to have pneumonia. Expectorate was collected by ejection into 30-ml antiseptic sputum-collection containers (Salivette, SARSTEDT, Nümbrecht, Germany). The ejections, as well as the tracheal and bronchial secretions, were Gram stained and examined by light microscopy at 80–1,000-fold magnification in a minimum of five viewing fields, following the principles of Bartlett [18]. Three basic culture media were then prepared for the growth of commonly occurring, rapidly growing aerobic microbes.

### Blood cultures

A least 20 ml of blood were added to two special culture media, BACTEC Plus Aerobic/F and Plus Anaerobic/F medium (BD, Becton, Dickinson and Company, Heidelberg, Germany).

### Duration of hospital stay

The lengths of hospital stays were compared between patients with pneumonia caused by *S. marcescens* or *P. mirabilis*.

### Mortality

The number of fatalities during hospital stays was measured in the study population. The survival rates were calculated by the Kaplan–Meier method.

### Statistical analysis

The nominal variables were stated in percentages and continuous data were indicated as mean ± standard deviation (SD). Two-tailed tests were calculated. The results were executed at a 95 % confidence interval (CI) for the sex differentiation of inpatients suffering from pneumonia triggered by *S. marcescens* or *P. mirabilis*. A chi-square test for two free variables of three possibilities was calculated on the VassarStats website for statistical calculation to determine whether *S. marcescens* and *P. mirabilis* were sensitive, intermediate, or resistant to antimicrobial agents, and to calculate acquisitions of pneumonia. A chi-square test was performed for two free variables of two possibilities for gender differences and deaths, and another chi-square test was performed for two free variables of five possibilities for specimens [19]. One-way analysis of variance (ANOVA) for two independent samples was performed to compare age differences, duration of hospital stay, and laboratory tests of patients with pneumonia caused by *S. marcescens* or *P. mirabilis.* Statistical significance was defined as a *P* value of less than 0.05.

### Ethics statement

The procedures of this retrospective study were performed in agreement with the established official guidelines of Witten/Herdecke University. All patient data were anonymized before evaluation. The Ethics Committee of Witten/Herdecke University approved this study protocol. Due to the retrospective nature of the study protocol, the committee waived the need for written informed consent.

## Results

A total of 6,932 patients of all ages were found with pneumonia triggered by diverse kinds of microorganisms at the HELIOS Clinic at Witten/Herdecke University in Wuppertal, Germany at the time of this clinical study from January 1, 2004, to August 12, 2014. Of these, 115 (1.7 %, 95 % CI 1.4 %–2.0 %) inpatients had pneumonia triggered by Gram-negative bacteria (ICD J15.6). A total of 43 (37.4 %, 95 % CI 28.6 %–46.2 %) inpatients were identified for this investigation as having pneumonia triggered by *S. marcescens*, and 20 (17.4 %, 95 % CI 10.5 %–24.3 %) inpatients had pneumonia due to *P. mirabilis*. Males were more frequently diagnosed with pneumonia triggered by *S. marcescens* or *P. mirabilis* (Table 1). No gender difference was found when comparing patients with pneumonia caused by *S. marcescens* or *P. mirabilis* (Table 1). *S. marcescens* and *P. mirabilis* infections were responsible for the most nosocomial-acquired pneumonia (Table 1). Gram-negative bacteria were discovered in most tracheal secretions in both groups of patients infected by these two organisms (Table 1). The duration of hospital stays did not differ between the two groups (Table 1).

**Table 1:** Demographic data, acquisition of pneumonia, length of hospital stay, mortality rate, and different discovery methods of *Serratia marcescens* and *Proteus mirabilis* infection in patients with pneumonia

Total No. of Gram-negative bacteria pneumonia = 115	*Serratia marcescens* (%)	*Proteus mirabilis* (%)	*P* value
No. of patients	43 (37.4)	20 (17.4)	
Gender			
Male	26 (60.5)	14 (70)	0.655
Female	17 (39.5)	6 (30)	0.655
Age mean + SD (years)	66.2 ± 13.4	64.6 ± 12.8	0.648
Acquisition of pneumonia			
Community-acquired pneumonia	15 (34.9)	6 (30)	0.083
Nosocomial-acquired pneumonia	23 (53.5)	7 (35)	0.083
Aspiration pneumonia	5 (11.6)	7 (35)	0.083
Specimens			
Tracheal secretions	33 (76.7)	9 (45)	0.056
Bronchial secretions	7 (16.3)	5 (25)	0.056
Sputum	1 (2.3)	2 (10)	0.056
Throat swab	0	2 (10)	0.056
Venous blood culture	2 (4.7)	2 (10)	0.056
Duration of hospital stay mean ± SD (days)	22.2 ± 18.1	18.4 ± 13.4	0.392
No. of deaths	7 (16.3)	3 (15)	0.807

Abbreviations: *SD* standard deviation

**Fig. 1:**
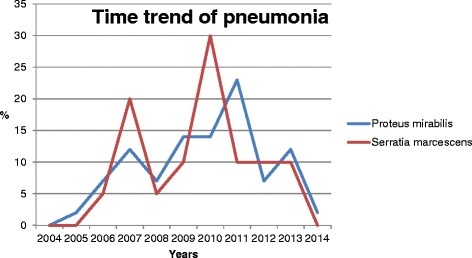
Time trend of pneumonia due to *Serratia marcescens* and *Proteus mirabilis* from 2004 to 2014

An increase in cases of pneumonia was found over the years of the study period (Fig. 1). The peak number of cases of pneumonia due to *S. marcescens* occurred in 2010 and due to *P. mirabilis* in 2011 (Fig. 1).

Two kinds of Gram-negative bacteria were discovered in the same patient in 9 cases with pneumonia. The other Gram-negative bacteria, apart from *S. marcescens* (43) and *P. mirabilis* (20), causing pneumonia during this study period were *Escherichia coli* (10 cases), *Enterobacter cloacae* (7), *Stenotrophomonas maltophilia* (6), *Acinetobacter baumannii* (5), *Enterobacter aerogenes* (3), *Citrobacter koseri* (1), *Moraxella catarrhalis* (1), *Prevotella buccae* (1), *Proteus vulgaris* (1), *Citrobacter koseri* (1), and *Serratia plymuthica* (1).

Fifty-two inpatients were excluded from this investigation due to infectious diseases triggered by other Gram-negative bacteria (37) or found in other locations in the body (15), or because of a lack of access to their records on the wards of the neurological department. In addition, all children and adolescents under the age of 18 with pneumonia triggered by *S. marcescens* or *P. mirabilis*, and who were hospitalized on the wards of the Division of Child and Adolescent Health, were excluded.

The number of examinations for each antimicrobial agent differed in this investigation because a few isolates were tested according to the CLSI guidelines, while others were tested corresponding to the EUCAST guidelines over the past few years. In general, the sums of the susceptibility tests were greater for the antibiograms obtained according to the CLSI guidelines (Tables 2 and 3).

**Table 2:** Antibiotic sensitivity and antibiotic resistance for different drug groups in patients with pneumonia caused by *Serratia marcescens*

No. of patients with *S. marcescens* = 43							
Drug groups	Active substance	No. using antibiotics (%)	No. of tests of antibiotics on antibiogram (%)	Sensitive (%)	Intermediate (%)	Resistant (%)	*P* value compared to ampicillin
Penicillins	Ampicillin	0	43 (100)	0	0	43 (100)	
	Piperacillin	0	43 (100)	31 (72.1)	5 (11.6)	7 (16.3)	**<** **0.0001**
Penicillin + Beta-lactamase inhibitors	Ampicillin + Sulbactam	3 (7.0)	43 (100)	0	0	43 (100)	1.0
	Piperacillin + Tazobactam	21 (48.8)	43 (100)	38 (88.3)	1 (2.3)	4 (9.3)	**<** **0.0001**
Cephalosporins	Cefepime	2 (4.7)	39 (90.7)	39 (100)	0	0	**<** **0.0001**
	Cefotaxime	0	43 (100)	36 (83.7)	2 (4.7)	5 (11.6)	**<** **0.0001**
	Ceftazidime	1 (2.3)	39 (90.7)	39 (100)	0	0	**<** **0.0001**
	Cefuroxime	4 (9.3)	43 (100)	0	0	43 (100)	1.0
Glycylcycline	Tetracycline	0	17 (39.5)	3 (17.6)	2 (11.8)	12 (70.6)	**0.001**
	Tigecycline	0	5 (11.6)	3 (60.0)	2 (40.0)	0	**<** **0.0001**
Carbapenems	Imipenem	5 (11.6)	43 (100)	42 (97.7)	0	1 (2.3)	**<** **0.0001**
	Meropenem	2 (4.7)	42 (97.7)	41 (97.6)	1 (2.4)	0	**<** **0.0001**
Gyrase inhibitors	Ciprofloxacin	4 (9.3)	42 (97.7)	37 (88.1)	2 (4.8)	3 (7.1)	**<** **0.0001**
	Levofloxacin	3 (7.0)	28 (65.1)	26 (92.9)	0	2 (7.1)	**<** **0.0001**
Aminoglycoside	Amikacin	0	26 (60.5)	18 (69.2)	8 (30.8)	0	**<** **0.0001**
	Gentamicin	2 (4.7)	40 (93.0)	38 (95.0)	2 (5.0)	0	**<** **0.0001**
	Tobramycin	0	27 (62.8)	16 (59.3)	4 (14.8)	7 (25.9)	**<** **0.0001**
Trimethoprim + Sulfamethoxazole	Co-trimoxazole	0	42 (97.7)	39 (92.9)	0	3 (7.1)	**<** **0.0001**
Others	Fosfomycin	0	17 (39.5)	17 (100)	0	0	**<** **0.0001**

Note: Significant *P* values shown in bold

**Table 3:** Antibiotic sensitivity and antibiotic resistance in different drug groups in patients with pneumonia caused by *Proteus mirabilis*

No. of patients with *P. mirabilis* = 20							
Drug groups	Active substance	No. using antibiotics (%)	No. of tests of antibiotics on antibiogram (%)	Sensitive (%)	Intermediate (%)	Resistant (%)	*P* value compared to ampicillin
Penicillins	Ampicillin	0	20 (100)	8 (40.0)	1 (5.0)	11 (55.0)	
	Piperacillin	1 (5.0)	20 (100)	13 (65.0)	4 (20.0)	3 (15.0)	0.023
Penicillin + Beta-lactamase inhibitors	Ampicillin + Sulbactam	3 (15.0)	20 (100)	13 (65.0)	3 (15.0)	4 (20.0)	0.065
	Piperacillin + Tazobactam	15 (75.0)	20 (100)	20 (100)	0	0	**0.0002**
Cephalosporins	Cefepime	0	19 (95.0)	19 (100)	0	0	**0.0003**
	Cefotaxime	0	20 (100)	18 (90.0)	0	2 (10.0)	**0.004**
	Ceftazidime	1 (5.0)	16 (80.0)	16 (100)	0	0	**0.0007**
	Cefuroxime	0	20 (100)	15 (75.0)	0	5 (25.0)	0.068
Glycylcycline	Tetracycline	0	13 (65.0)	0	0	13 (100)	**0.018**
	Tigecycline	0	1 (5.0)	1 (100)	0	0	0.497
Carbapenems	Imipenem	3 (15.0)	20 (100)	19 (95.0)	1(5.0)	0	**0.0004**
	Meropenem	0	19 (95.0)	18 (94.7)	1 (5.3)	0	**0.0006**
Gyrase inhibitors	Ciprofloxacin	1 (5.0)	18 (90.0)	15 (83.3)	1 (5.6)	2 (13.3)	**0.016**
	Levofloxacin	1 (5.0)	14 (70.0)	13 (92.9)	1 (7.1)	0	**0.003**
Aminoglycoside	Amikacin	0	9 (45.0)	9 (100)	0	0	**0.01**
	Gentamicin	2 (10.0)	19 (95.0)	16 (84.2)	0	3 (15.8)	0.080
	Tobramycin	0	11 (55.0)	8 (72.7)	1 (9.1)	2 (18.2)	0.139
Trimethoprim + Sulfamethoxazole	Co-trimoxazole	0	20 (100)	14 (70.0)	0	6 (30.0)	0.321
Others	Fosfomycin	0	5 (25.0)	5 (100)	0	0	0.056

Note: Significant *P* values shown in bold

The most-used antibiotic in patients with pneumonia due to *S. marcescens* or *P. mirabilis* in this research was piperacillin-tazobactam (Tables 2 and 3).

Among the inpatients with pneumonia triggered by *S. marcescens* or *P. mirabilis*, no resistance was found to cefepime when compared to ampicillin; this finding was statistically significant (Tables 2 and 3). *S. marcescens* and *P. mirabilis* had the highest antibiotic-resistance rates toward ampicillin, compared to cefepime, in this research (Tables 2 and 3). *S. marcescens* also had an elevated antibiotic-resistance rate toward ampicillin, as correlated with the ampicillin-sulbactam combination used in this study (Table 2).

The mortality rate did not statistically differ between the study groups. The survival rate was 83.7 % (95 % CI 71.7 %–95.8 %) in the study population with pneumonia triggered by *S. marcescens* and 85.0 % (95 % CI 68.0 %–102.0 %) in in the group infected with *P. mirabilis* (Table 1).

## Discussion

In general, *S. marcescens* and *P. mirabilis* are bacterial strains that are not frequently found in pneumonia patients, as was also the case in the present study. No antibiotic resistance developed to cefepime—an antimicrobial agent administered to patients with pneumonia—in either *S. marcescens* or *P. mirabilis* during this ten-year, qualitative, controlled observational investigation. An earlier clinical study comparing the effectiveness of cefepime to six other antibiotics commonly used in the management of severe infections triggered by Gram-negative pathogens indicated that the most effective antibiotics were cefepime and imipenem [20]. A comparable result was found in another investigation that evaluated cefepime as an initial antibiotic treatment for Gram-negative pneumonia. A previous study had compared beta-lactam antibiotics against all Gram-negative pathogens that cause pneumonia, and based on that study’s outcome, cefepime was suggested as an initial therapy for nosocomial-acquired pneumonia triggered by Gram-negative pathogens [21].

Similar resistance rates were found for amikacin and fosfomycin in the present work. Note, however, that the sensitivities of these two antibiotics were significantly lower than that of cefepime in this investigation. Amikacin has known effectiveness against Gram-negative bacteria [22].

A previous retrospective study that investigated the optimal first-antibiotic regimen for nosocomial-acquired pneumonia triggered by Gram-negative bacteria detected in sputum [23] indicated good effectiveness with imipenem (75 %) and amikacin (84 %), based on antibiogram results. In comparison, the sensitivity rate determined in the present study was higher for imipenem (97.7 %) and considerably lower for amikacin (69.2 %). The earlier study took *Pseudomonas* into account in the evaluation of the Gram-negative bacteria, but a direct comparison of studies is often complicated because different conspecifics were investigated under the collective term “Gram-negative bacteria” [23].

The clinical efficacy of cefepime was previously confirmed by comparative and non-comparative studies conducted many years ago [23, 24]. Cefepime was considered useful in the management of pneumonia, and it is also active against organisms that show resistance to other drugs [23, 24]. Fosfomycin is widely used for nosocomial-acquired infections in hospitals because of its good efficacy against Gram-negative bacteria [25, 26]. It can also be used in infections of the airways [27]. Meropenem is a broad-range antimicrobial agent effective against Gram-positive and Gram-negative germs [28] and has shown excellent efficacy in clinical studies for the management of severe pneumonia in critical patients [29]. Meropenem also showed good effectiveness and relatively low resistance in all inpatients with pneumonia due to *S. marcescens* and *P. mirabilis* over the course of the present clinical research.

Good results were also found for levofloxacin in a previous study [29]. Levofloxacin was likewise found to be effective in the present study for the management of patients with pneumonia triggered by *S. marcescens* or *P. mirabilis*. The development of resistance to levofloxacin was relatively low over the 10-year period covered by the present investigation. It has good bacteriological effectiveness against a variety of infection diseases, and it is also approved for the antibiotic management of nosocomial-acquired infections [30].

When compared to levofloxacin, imipenem showed better efficacy and a somewhat poorer resistance rate in the present clinical study of hospitalized patients with pneumonia triggered by *S. marcescens* or *P. mirabilis*. Another study arrived at the same conclusion, where levofloxacin was as effective as imipenem and was tolerated well in patients with nosocomial-acquired pneumonia [31].

A nationwide study conducted in Japan concluded that ciprofloxacin had favorable susceptibility rates for treatment of Gram-negative bacterial pneumonia [32], in agreement with the findings of the present study. Another study that examined the effect of intravenous administration of ciprofloxacin on nosocomial-acquired pneumonia proposed intravenous ciprofloxacin as the first choice for nosocomial-acquired pneumonia triggered by both Gram-positive and Gram-negative microbes [33].

Gentamicin is mostly effective against Gram-negative microorganisms and shows activity against staphylococci [34]. It had relatively good activity in the present study in patients with pneumonia triggered by *S. marcescens* or *P. mirabilis*. Both *S. marcescens* and *P. mirabilis* showed resistance to gentamicin.

Ceftazidime is a valuable alternative for the management of nosocomial-acquired pneumonia; nevertheless, its role as an effective antimicrobial agent has decreased over the past decade due to the strong increase in resistance rates, especially for *Pseudomonas aeruginosa* and *Acinetobacter baumannii* [35]. *Pseudomonas* was not the focus of the investigation in the present research, but an increasing development of antibiotic resistance of *S. marcescens* and *P. mirabilis* against ceftazidime was observed. Clinical studies have shown that the beta-lactam inhibitor combination of piperacillin-tazobactam is an effective medication for patients suffering from nosocomial-acquired pneumonia [36]. Piperacillin-tazobactam and cefepime showed similar effectiveness against Gram-negative bacteria, but *S. marcescens* and *P. mirabilis* developed greater resistance to piperacillin-tazobactam in the present study. Due to this elevated resistance, piperacillin-tazobactam represents an essential support option in the treatment of nosocomial infections.

Resistance to tobramycin can be noted in numerous stages and is generally high due to the preservation of aminoglycoside-modifying enzymes [37]. Tobramycin showed increasing resistance rates in *S. marcescens and P. mirabilis* in the present study in hospitalized patients with pneumonia. Tobramycin was not as commonly tested in this study, but its efficacy was reduced in the antibiograms of the hospitalized patients with pneumonia.

Co-trimoxazole (a combination of trimethoprim and sulfamethoxazole) was effective in the study groups with pneumonia due to *S. marcescens* or *P. mirabilis*, but Gram-negative bacteria also showed significant resistance to co-trimoxazole in the present study. The range of effectiveness of the co-trimoxazole combination favors eradication of Gram-positive and Gram-negative microorganisms, although it is also effective against protozoa and some types of fungi [38].

Cefotaxime acts against Gram-positive and Gram-negative microbes, but elevated antibiotic resistance of *S. marcescens and P. mirabilis* to cefotaxime was evident in the present study population [39].

Tetracyclines are bacteriostatic toward Gram-positive and Gram-negative microbes [40]. Despite the description of good efficacy of tetracycline in the medical literature, it was poorly effective against *S. marcescens* and *P. mirabilis* pneumonia in the present investigation and increased development of resistance was apparent in these bacteria.

Piperacillin has the broadest range of activity of all penicillins toward Gram-positive and Gram-negative microorganisms, including *Pseudomonas* and *Enterobacteriaceae* [41]. Piperacillin was significantly more effective against Gram-negative isolates in patients with pneumonia than were the other representatives of penicillin, but *S. marcescens* and *P. mirabilis* showed resistance to it in the present investigation. Piperacillin was more effective against *S. marcescens* and *P. mirabilis*-associated pneumonia in the present study when combined with beta-lactamase inhibitors.

Cefuroxime has increased activity against Gram-negative rods when compared to first-generation cephalosporins. It also shows high stability against beta-lactamases [42]. Therefore, cefuroxime can be used for the initial therapy of pneumonia due to beta-lactamase-producing strains. In the current period of rapidly growing bacterial resistance, the appropriate use of new antibacterial agents would be favored in the empirical treatment of pneumonia [43]. Due to the resistance to cefuroxime apparent in the antibiograms of isolates from tracheal secretions of inpatients with pneumonia in the present investigation, an empirical treatment would not be recommended for suspected pneumonia due to *S. marcescens* or *P. mirabilis*.

Ampicillin is effective against Gram-positive and some Gram-negative microorganisms; therefore, it is called a broad-range antimicrobial agent [44]. However, Gram-negative bacteria in hospitalized patients suffering from pneumonia showed the strongest resistance to ampicillin in the present investigation. The susceptibility testing indicated by the antibiograms of isolates recovered from inpatients with pneumonia in this clinical research indicated that *S. marcescens* and *P. mirabilis* were resistant to ampicillin, as expected. Ampicillin in combination with beta-lactam inhibitors showed better results, based on the susceptibility testing of tracheal secretions from inpatients with pneumonia triggered by *S. marcescens* or *P. mirabilis* in this study. Ampicillin-sulbactam has proven to be an important antimicrobial agent in the therapeutic arsenal for the effective treatment of pneumonia [45].

Antibiotic resistance in pathogens causing respiratory tract infections has increased dramatically in recent years. Resistance to penicillins due to beta-lactamase production has become a widespread problem around the world [46]. The careful selection of antibiotics with low potential for resistance, in addition to actively working against the further development of penicillin resistance, is the best current strategy.

### Study limitations

This clinical research explains the state of antibiotic resistance in inpatients with pneumonia triggered by *S. marcescens* or *P. mirabilis* in a single teaching hospital, so the study results cannot be related to different geographical regions. Interpretation of the study data revealed that not all of the antimicrobial agents were examined in equal quantities in the susceptibility testing of inpatients with pneumonia triggered by *S. marcescens* or *P. mirabilis*. The authors were not able to clarify whether all the antimicrobial agents were analyzed for each isolate of *S. marcescens* and *P. mirabilis*.

### Conclusions

All the study patients with pneumonia due to *S. marcescens* or *P. mirabilis* presented antibiotic resistance to a diversity of antimicrobial agents, but none showed resistance to cefepime. All common antimicrobial agents must be examined for effectiveness in the event of detection of Gram-negative bacteria on agar plates, for all patients who are identified with pneumonia. This should be done both for the immediate antimicrobial management of patients with pneumonia triggered by Gram-negative bacteria and for observing the temporal formation of antibiotic resistance in Gram-negative pathogens in subsequent periods.

## Abbreviations

CLSI: clinical and Laboratory Standards Institute
CRP: c-reactive protein
CI: confidence interval
EUCAST: europe-wide standards for susceptibility testing
ICD: international Classification of Diseases
MALDI-TOF-MS: matrix assisted laser desorption ionization time-of-flight mass spectrometry
MIC: minimum inhibitory concentration
SD: standard deviation
